# Diatom Lensless Imaging Using Laser Scattering and Deep Learning

**DOI:** 10.1021/acsestwater.4c01186

**Published:** 2025-03-24

**Authors:** Ben Mills, Michalis N. Zervas, James A. Grant-Jacob

**Affiliations:** Optoelectronics Research Centre, University of Southampton, Southampton SO17 1BJ, U.K.

**Keywords:** diatoms, lasers, deep learning, scattering, lensless sensing

## Abstract

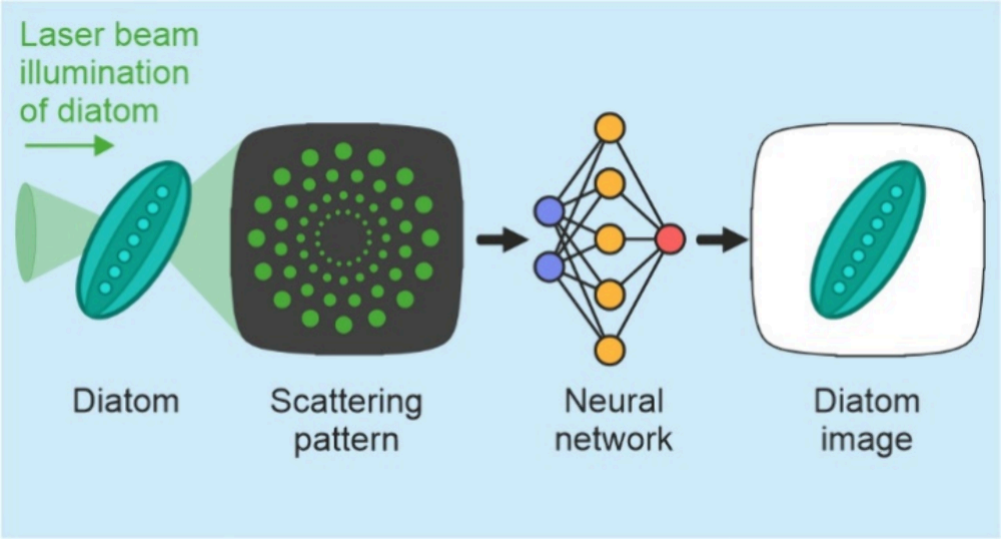

We present a novel approach for imaging diatoms using lensless imaging and deep learning. We used a laser beam to scatter off samples of diatomaceous earth (diatoms) and then recorded and transformed the scattered light into microscopy images of the diatoms. The predicted microscopy images gave an average SSIM of 0.98 and an average RMSE of 3.26 as compared to the experimental data. We also demonstrate the capability of determining the velocity and angle of movement of the diatoms from their scattering patterns as they were translated through the laser beam. This work shows the potential for imaging and identifying the movement of diatoms and other microsized organisms in situ within the marine environment. Implementing such a method for real-time image acquisition and analysis could enhance environmental management, including improving the early detection of harmful algal blooms.

## Abstract 2

Monitoring diatoms is important in understanding the health of the marine environment. This study documents the use of lensless sensing to image samples of diatoms and quantify their movement.

## 1 Introduction

Diatoms are a major group of algae, specifically microalgae, found in the oceans, waterways, and soils of the world.^1,2^ They are foundational to marine food webs and are unicellular organisms that form an integral part of the phytoplankton community. Diatoms exist in a range of sizes from 5 μm to 5 mm and are characterized by their unique silica cell walls, known as frustules, which exhibit complex patterns.^3^ These microorganisms are incredibly diverse, with estimates suggesting there are over 100,000 species globally.^4^

Diatoms play a crucial role in the environment as they form the base of the aquatic food web by converting carbon dioxide into organic carbon through photosynthesis, contributing to ∼20% of photosynthetically fixed CO_2_ on Earth.^5^ They are responsible for producing ∼20% of the world’s oxygen and have a rapid nutrient uptake,^6^ thus making them vital for both marine ecosystems and the planet’s overall health. In addition, certain types of algae can, under certain conditions, lead to harmful algal blooms (HABs), which can have a negative impact on marine aquaculture by generating toxins or very high levels of deoxygenation, or even by damaging gills due to high-density levels within the water.^7−9^

As such, the imaging and sensing of diatoms are critically important for understanding and monitoring HABs and can provide insights into environmental conditions, as their presence and abundance can indicate water quality and changes in ecological status.^10^ As diatoms are sensitive to factors such as fluctuations in temperature, CO_2_ concentration, and ocean acidification, they also serve as key indicators of the impact of climate change on aquatic ecosystems.^11^ Monitoring diatom populations is therefore essential for understanding and managing aquatic ecosystems, particularly in the face of climate change and pollution.

Diatoms' unique silica structures and sizes make them excellent subjects to image for monitoring via lab-based microscopy following sample collection^12,13^ or field-based microscopy and flow cytometry.^14,15^ However, such methods of diatom monitoring and imaging can be costly and bulky and, as such, require expensive oceanographic missions that are often small in number and infrequent, meaning that monitoring can have low spatial and temporal resolution. There is a clear need for low-cost and efficient sensors that can facilitate the widespread and continuous monitoring of diatom populations. Whilst methods to reduce the microscopy costs to under $400 and enable citizen science have been explored,^16,17^ such technology is still too bulky and expensive for mass deployment.

Digital holography^18^ is an imaging technique for recording and reconstructing three-dimensional images by capturing the interference pattern between an object beam and a reference beam, using digital sensors such as CMOS cameras. This method enables high-resolution, detailed analysis of submillimeter-sized particles and biological samples but requires precise optical alignment.

Underwater digital holography of marine plankton has been explored using a subsea digital holographic camera (eHoloCam) for analyzing and identifying marine organisms and particles.^19^ Digital holograms were recorded on an electronic sensor and reconstructed numerically, offering advantages such as three-dimensional spatial reconstruction and nonintrusive sampling. The paper presents images from deployments in the North Sea and the Faeroes Channel.

Similarly, Dyomin et al.^20^ highlighted the use of submersible holographic cameras for in situ plankton studies for noninvasive, real-time monitoring of plankton size, shape, and behavior. The study emphasized the importance of Fourier spectra in bioindication, showing how plankton behavior and environmental changes can be detected early through spectroscopic analysis, aiding in ecosystem health assessment and early pollution detection.

Additionally, a submersible holography system for in situ recordings of plankton distribution has been described by Malkiel et al.^21^ By employing a ruby laser with an inline recording configuration, the system captured high-resolution images of plankton, revealing variations in plankton population between different water layers, particle concentration maxima near a pycnocline, and evidence of zooplankton migration.

In contrast to digital holography, lensless sensing reduces the requirements of imaging optics,^22^ thus reducing both cost and complexity, and also enables real-time processing and robust performance even in noisy, low-contrast environments, hence unlocking the potential for the development of portable devices for widespread deployment. Lensless sensing computationally transforms the pattern of light that is scattered and imaged from an object into an image of the object itself, in a process commonly known as phase retrieval.^23^ However, conventional phase retrieval algorithms can be slow as they are iterative processes,^24^ and therefore, alternative single-step methods need to be explored to unlock real-time lensless imaging.

The emergence of deep learning neural networks has revolutionized the field of image analysis, with convolutional neural networks (CNNs) having the ability to rapidly identify and label a large amount of data.^25^ CNNs have been used for identification of plant species from leaf photographs,^26^ pollen grains,^27^ and feature labeling in placenta scanning electron microscopy (SEM) images.^28^ Specifically related to this work, by automating the identification and classification of diatoms, deep learning models can process vast data sets quickly and accurately,^29,30^ which is essential for monitoring marine ecosystems. One of the most impactful applications of deep learning in this domain is the use of image-to-image algorithms,^31,32^ such as conditional generative adversarial networks (cGANs). These algorithms can transform images from one domain to another, such as one modality to another, enhancing the quality and resolution of objects^33,34^ and enabling the detailed analysis of structures. Applications of this approach also include holography,^35,36^ ptychography,^37^ and transforming scattering patterns into images of pollen grains.^38^ Unlike pollen grains, the shape and size of diatoms can vary strongly, and they can scatter light more strongly due to their high-contrast periodic structures.^39^ Here, we use an image-to-image neural network to transform scattering patterns of samples of diatomaceous earth into microscopy images of samples. We also use neural networks to identify the velocity and angle with which they are translated, as this could enable understanding and monitoring of the environment in which they are detected. Importantly, as this neural network approach is single-step, this approach could be implemented in real-time and in situ. This lensless imaging technique allows for the potential of smaller footprint, low-cost sensors to be developed and distributed in the marine environment.

## 2 Experimental Methods

### 2.1 Sample Preparation

Diatomaceous earth (written as diatoms henceforth) was dispersed onto a 25 × 75 × 1 mm thick soda-lime glass slide (Corning) using a laboratory-grade cotton bud. The slide was tapped onto the surface of a worktop so that any nonadhering diatoms were removed from the slide.

### 2.2 Experimental Setup

**Figure 1:**
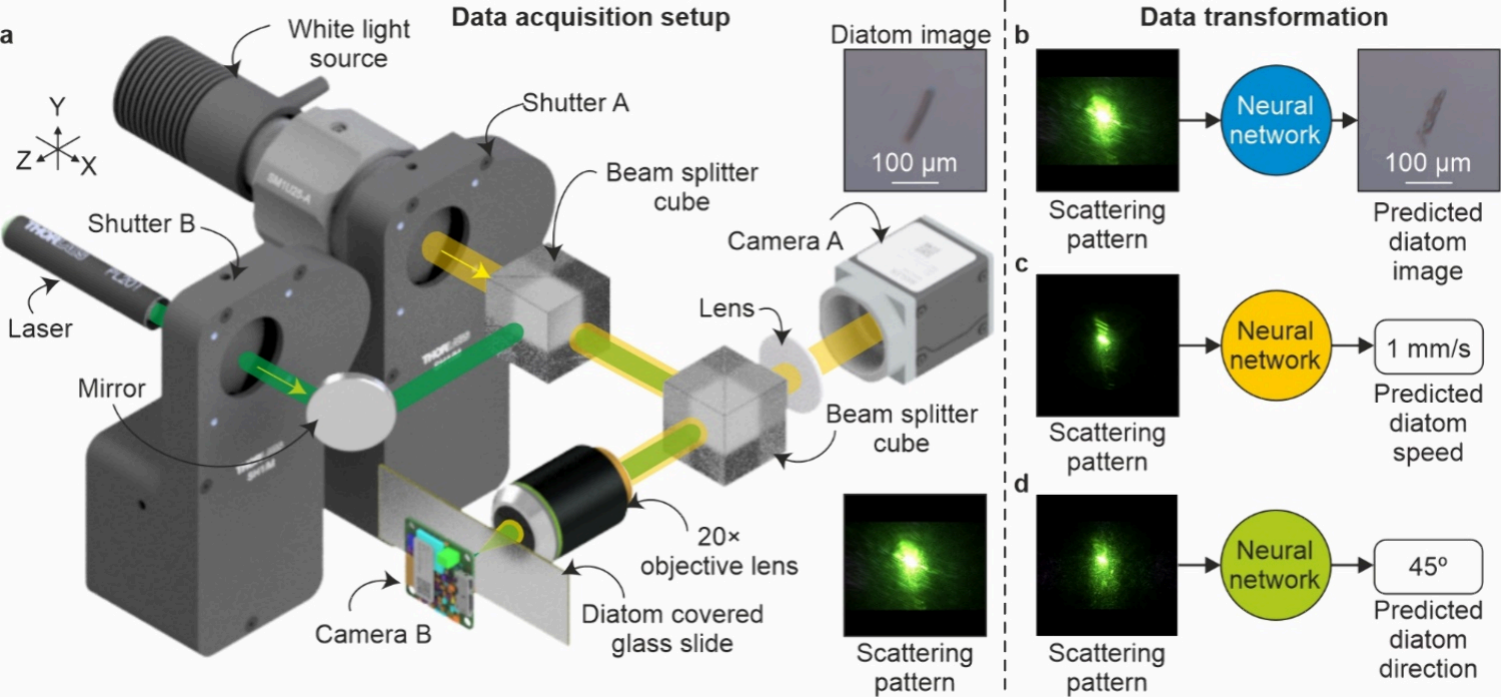
(a) Diagram of the experimental setup used to simultaneously image the diatoms and capture their scattering pattern from laser illumination. Three neural networks employed in this work were (b) image generation, (c) speed classification, and (d) angular direction classification.

As demonstrated in the diagram in Figure 1a, a laser diode with a 520 nm central wavelength (green light) and a 4.5 mW output power with a collimated output (Thorlabs, PL203) was focused onto the surface of the diatom sample using a 20× objective (Olympus, LMPLFLN). This objective also allowed simultaneous imaging of the diatom sample via illumination using a 570 mW white-light LED (Thorlabs, MWWHL4) and the use of a camera (Basler, daA1920-160uc, 1920 × 1200 pixels, RGB, Camera A). The illumination of the sample via the white-light LED was blocked via a shutter (Thorlabs, SH1, shutter A) so that only forward scattered green light from the diatoms was collected by a bare board camera (Basler, daA1920-160uc, 1920 × 1200 pixels, RGB, camera B) for collecting the scattering patterns, whilst another shutter (Thorlabs, SH1, shutter B) enabled blocking of the laser for collecting the microscopy images. The laser spot size was 50 μm on the glass slide surface. The sample was mounted on motorized stages (Zaber, X-LSM050A-E03, X-LSM100A, X-VSR20A-E01) to allow for movement in the *XYZ* direction, where *Z* is the laser axis, and *XY* is the plane parallel to the surface of the sample.

### 2.3 Data Collection

Data were acquired using a Dell Precision 7865 Windows 10 workstation consisting of an Intel(R) Xeon(R) Gold 5222 CPU at 3.80 and 3.79 GHz (2 processors) that had three NVIDIA A4500 GPUs (20 GB VRAM each). Python code running on the workstation was used to automate the data collection via controlling the XYZ stages and the shutters, and capturing the images from the cameras. For each of the 149 diatoms imaged for the microscopy neural network, the stages were translated in a spiral pattern from the initial center so that 9 images at different positions ±5 μm (in the *XY* plane) around the laser focus were acquired to provide the neural network with more information than would be achieved from just a single scattering pattern to account for any inhomogeneity in the laser spatial intensity profile at the focus. The diatom microscope images were cropped to 256 × 256 pixels, whilst the scattering pattern images were padded with zeros to form a square image (for neural network training) and to keep the high spatial frequency information at the edges of the images and were then cropped and resized to 256 × 256 pixels before being used for training the microscopy neural network. The imaging camera (camera A) and scattering camera (camera B) had integration times of 300 ms and 500 μs, respectively.

For both the velocity and angle data collection, an exposure time of 100 ms for camera B was used to allow for enough scattering data to be acquired, while the diatom was translated through the beam. In this instance, to allow for a longer integration time on the scattering camera, a neutral density filter of 3 was used to avoid oversaturation of the images. The images for both the varying velocity and the varying angle were cropped into 1200 × 1200 pixel squares around the central region and then resized to 512 × 512 pixels for training and testing. Padding was not included in these images. To capture data on the varying velocity of the diatoms, the diatoms were translated through the focus by 50 μm in the *X*-direction. Scattering patterns from 42 different diatoms translated at 10 different velocities (0.1 to 1 mm/s, in steps of 0.1 mm/s). In addition, scattering patterns were recorded while translating the diatoms at different angles from 0° to 345° in steps of 15° at a velocity of 0.2 mm/s. Scattering patterns from 28 different diatoms were captured (excluding 165° and 180°). Data for 165° and 180° (10× each) were left out of the training data to test the capability of the neural network to predict angles not present in training.

### 2.4 Neural Networks

The scattering patterns were paired with the microscopy images for training a cGAN using an architecture known as Pix2pix. The training was carried out using the same workstation that was used to acquire the data. A total of 1283 diatom images and scattering patterns were used to train the neural network that had a 7-layer architecture (see, for example, ref (40)), a learning rate of 0.0002, and a drop-out of 0.5. The neural network was trained for 100 epochs (8 h) until the training loss errors reached a minimum. Once trained, the neural network was applied to scattering patterns not used in training, and the output (predicted images of diatoms) was compared to the experimentally obtained diatom images.

For determining the speed of the diatoms as they were translated through the laser focus, a regression CNN was used that comprised 28 layers, consisting of 7 convolutional layers (each followed by batch normalization and ReLU activation), 3 pooling layers, one drop-out layer, a fully connected layer, and a regression output layer. The neural network was trained on velocities of 0.1 to 1 mm/s in steps of 0.1 mm/s. An 85:15:5% training/validation/testing split of the data was used for training the neural network, meaning 336 images were used for training, 59 images for validation, and 25 images for testing. The neural network was trained for 25 epochs (∼8 min), which was the time in which the loss and RMSE (root-mean-square error, i.e., the difference between the actual and predicted values) had plateaued.

In addition, another regression neural network of the same structure was used to determine the angle of direction at which the diatoms were moving when translated for 25 μm from the center of the laser beam focus at different angles. A split of the data was used for training the neural network, meaning 525 images were used for training, 49 images for validation, and 43 images for testing. This time the neural network was trained for 5 epochs, which took ∼3 min.

## 3 Results and Discussion

**Figure 2:**
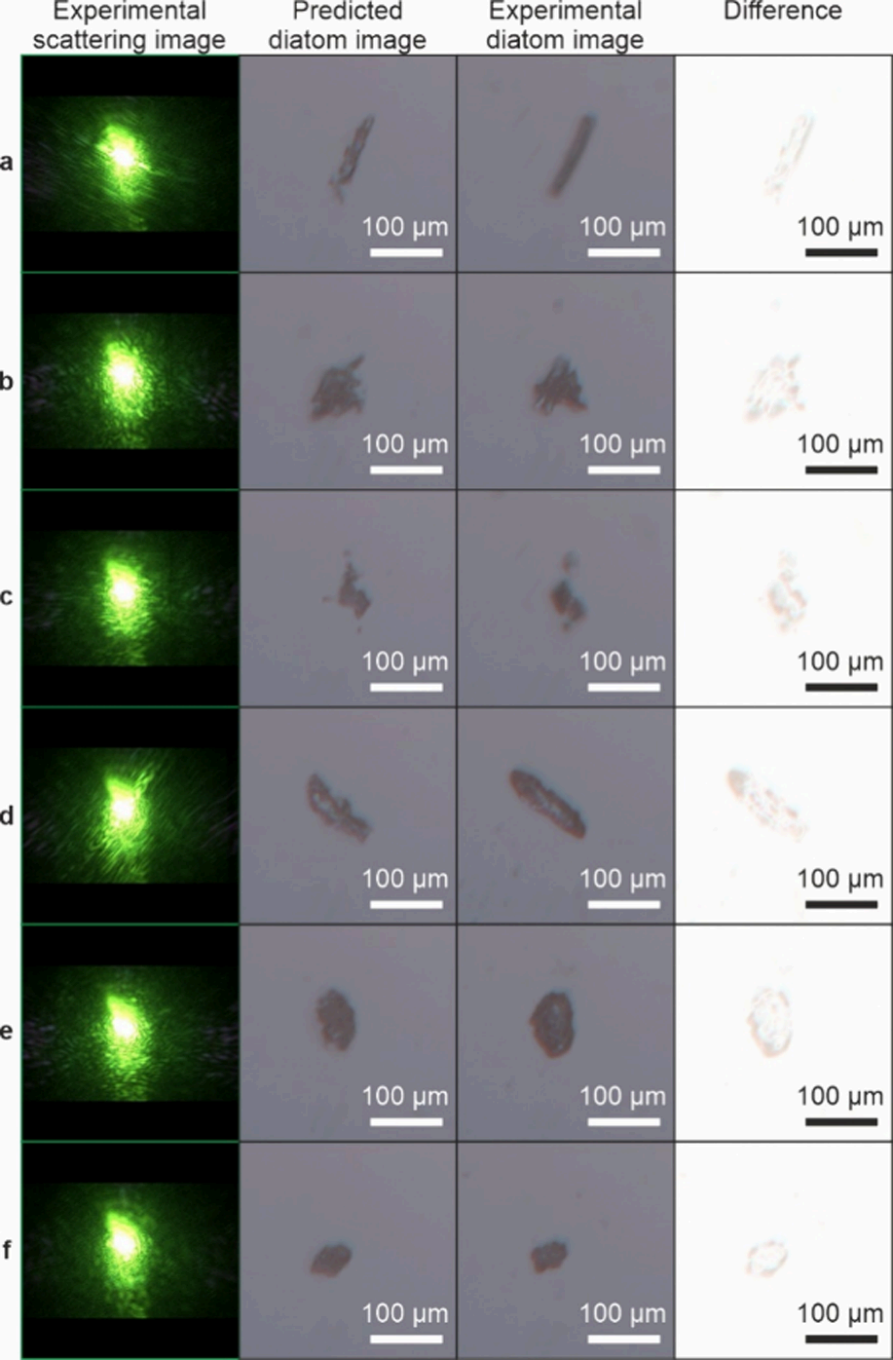
Experimental diatom scattering patterns (column 1) and the associated neural network predicted images (column 2). Also included are the corresponding experimental images (column 3) and the difference between the two images (column 4) (one minus the other such that white pixels indicate less difference).

Figure 2 shows the test data consisting of 6 different diatoms, with column 1 showing the experimental scattering pattern, column 2 showing the predicted diatom image, column 3 showing the experimental diatom image, and column 4 showing the difference in the images, such that lighter pixels indicate regions of less difference.

The predicted images in Figure 2 were evaluated by using the peak signal-to-noise ratio (PSNR), where a higher value means a more accurate predicted image. We also used the structural similarity index measure (SSIM), in which a value of 1 means the predicted and experimental images are the same, 0 indicates there is no similarity, and −1 indicates the pairs of images are completely anticorrelated, the RMSE, where a smaller value means greater similarity, and the perceptual image quality evaluator (PIQE), which provides a metric based on perceptual image quality (a smaller score indicates better perceptual quality). These image metrics are displayed in Table 1 and are calculated using the equations below.

**Table 1:** PSNR, SSIM, RMSE, and PIQE for the Predicted and Experimental Pollen Images Shown in Figure 2

image	PSNR	SSIM	RMSE	PIQE
a	40.60	0.98	2.38	45.89
b	37.25	0.98	3.50	48.85
c	36.75	0.98	3.71	46.95
d	35.75	0.98	4.16	47.62
e	37.15	0.98	3.54	46.53
f	40.89	0.99	2.30	45.75
average	38.07	0.98	3.26	46.93

The PSNR of all the predicted images was calculated as follows:
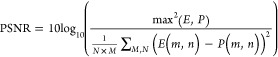
such that *M* and *N* represent the total number of rows and columns of pixels in the images, respectively, and *m* and *n* denote specific pixel positions within each row and column, respectively. The term max(*E*,*P*) is the maximum intensity value present in either the experimental image *E* or the predicted image *P*.

The SSIM is a metric that measures the luminance, contrast, and structural similarity. For each pair of experimental and predicted images, the SSIM was computed using the following equation:
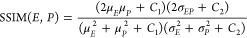
where μ_*P*_ is the mean of *P*, μ_*E*_ is the mean of *E*, σ_*P*_^2^ is the variance of *P*, σ_*E*_^2^ is the variance of *E*, and σ_*EP*_ is the covariance of *E* and *P*. *C*_1_ = (0.01*L*)^2^ and *C*_2_ = (0.03*L*)^2^, respectively, and *L* denotes the dynamic range of the pixel values in the images.

The RMSE is an error metric that quantifies the average difference between predicted and actual image pixel values. To calculate the RMSE, the squared differences between the corresponding pixel intensity values in the predicted and experimental images are averaged, and the square root of this mean is taken. The RMSE is calculated using the formula:
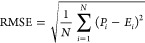
where *E*_*i*_ is the experimental image pixel value, *P*_*i*_ is the predicted image pixel value, and *N* is the number of pixels.

Finally, PIQE is an image quality assessment algorithm designed to quantify perceptual quality without the need for a reference image. It operates by dividing the input image into nonoverlapping blocks and analyzing each block for distortions. The algorithm estimates blockwise distortion and measures the local variance of perceptibly distorted blocks to compute the quality score. The final PIQE score ranges from 0 to 100, with lower scores indicating better perceptual quality. The PIQE score is interpreted as follows: 0–20: excellent quality, 21–35: good quality, 36–50: fair quality, 51–80: poor quality, and 81–100: bad quality.^41^

As shown in Table 1, the SSIM values for all images are approximately the same high value, either 0.98 or 0.99, indicating good agreement with the experimental images. From the values in the table, all images have similar metrics, with Figure 2f being the most accurately predicted and Figure 2d being the least accurate in terms of RMSE and PIQE. The RMSE for all images is below 5, indicating that the average error per pixel is only about 2% of the full intensity range. This suggests that the predicted images are very close to the experimental images, demonstrating high accuracy in the reconstruction process. Indeed, from the figures, it is evident that the predicted images are visually similar to the experimental images. All images have a PIQE value that can be considered to have fair quality. Further improvements to the training data (i.e., more varied data) or an alternative network architecture could lower these PIQE scores and enhance the overall perceptual quality.

**Figure 3:**
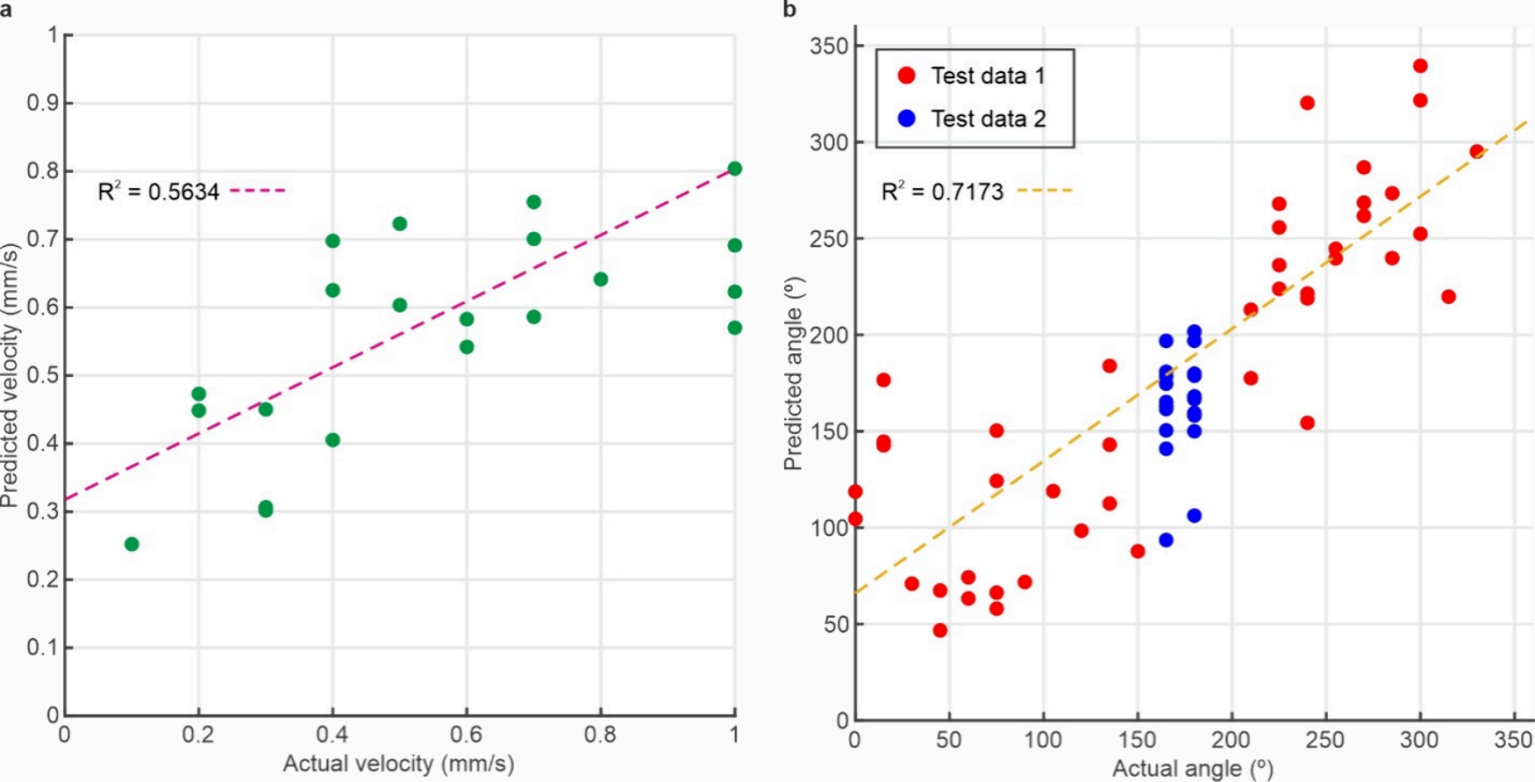
(a) Predicted diatom velocity compared with experimental velocity. (b) Predicted diatom angle compared with experimental angle when moving at a velocity of 0.2 mm/s. Test data 1 is data from angles used during training of the neural network, while test data 2 is data from angles not present in the training data.

The ability to determine the velocity of diatoms using a regression CNN is shown in Figure 3a, which displays the experimental velocity compared to the predicted velocity. The *R*-value of the plot is 0.5634, with the RMSE between the predicted and experimental data being 0.189 mm/s. The graph shows that higher experimental velocities are correlated with higher predicted velocities.

In addition, Figure 3b demonstrates the capability of a regression CNN to determine the angle of movement of a diatom, displaying a plot of the experimental angle compared with the predicted angle. The *R*-value of the plot is 0.7173, with an RMSE for the combined data of 49.55°.

The capability of the neural network to produce images and velocity and angle from the scattering patterns without prior data means that such a neural network could be implemented in real-time in situ, with only needing a laser, objective lens, and camera B and without shutters and an imaging camera. Such as setup could be further compacted with a simpler lens and could be deployed in the marine environment using a microcomputer, such as a Raspberry Pi,^42^ with appropriate housing.

The technique demonstrated here allows a neural network to generate the images of diatoms not previously included in the training data set, enabling the potential detection of unknown or rare species. However, to achieve reliable species-level identification, it may be necessary to incorporate higher-resolution data (e.g., SEM images^43^) and expand the training set to include broader morphological variations. Further to this, by expanding the data set to include a broader range of microorganisms, it could be possible to enhance the network’s ability to generalize and reconstruct new or rare species accurately.

Moreover, because cyanobacteria (typically 0.5–100 μm)^44^ and protozoa (typically 20–200 μm)^45^ share similar size ranges with diatoms, our approach can be extended to image these organisms as well. This could be useful in marine sensing since *Microcystis* is a genus of cyanobacteria known to indicate HABs,^46^ and protozoan pathogens like *Cryptosporidium* and *Giardia* are critical indicators for water safety.^47^ Including these in the training process would enable more diverse detection, aiding water biologists in real-time field analysis.

Additionally, neural networks such as segmentation models could be used alongside our imaging approach to distinguish individual species within the aggregated species. Similar segmentation strategies have recently been applied to detect overlapping algae^48^ and pollen grains.^49^ By implementing a segmentation network that can differentiate microorganisms from each other, microplastics, or other debris, we can improve the accuracy of biosurveillance and biodiversity assessments. Ultimately, broadening our data set and refining our network architectures will yield a more powerful, versatile tool for marine environmental monitoring, water safety, and microplastic detection in complex real-world settings.

In real-world environments, factors such as water turbidity, particle interference, and variations in background light levels could affect the accuracy of scattering-based imaging. Therefore, various methods to ensure that neural networks can accurately reconstruct images of diatoms should be explored. For example, varying the background light in the training data could help the neural network learn to reconstruct diatoms correctly, regardless of light levels. Similarly, using different optical filters or varying the turbidity or salinity of a volume of water between the diatom and the sensor during training data capture could improve the accuracy. Additionally, data augmentation techniques^50,51^ could be employed to artificially alter background lighting levels,^52^ modify the clarity of the scattering pattern images,^53^ and introduce additional artificial particulates or biofouling that may occlude the scattering pattern from the sensor.^54^ Such methodologies would not only enhance the robustness of the neural network but also reduce the need for diverse training conditions, thereby increasing the speed of data collection and training. Furthermore, implementing real-time adaptive algorithms that adjust to changing environmental conditions could further improve the reliability of the system in field applications.

## 4 Conclusions

We have demonstrated the capability of using deep learning to transform scattering patterns from laser-illuminated diatoms into 20× microscope objective images of diatoms. We tested the neural network on 50 different images, producing an average PSNR, SSIM, RMSE, and PIQE value of 38.07, 0.98, 3.26, and 46.93, respectively, with diatoms in the predicted images showing similarity in size and shape to their corresponding experimentally obtained microscopy images. We also showed the capability of neural networks to predict the velocity and angle of diatoms from their scattering patterns as they were translated through laser focus. This work shows the potential for using lensless sensing for imaging diatoms in the marine environment, which could allow the mass deployment of sensors that enable real-time imaging and thus monitoring of diatoms and their populations, potentially aiding in understanding environmental tipping points and harmful algae blooms. The technique demonstrated could also be applied to microplastic monitoring in a marine environment.

## Data Availability

The data underlying this study are openly available in University of Southampton Institutional Research Repository at https://doi.org/10.5258/SOTON/D3420.
